# Modified human glucagon-like peptide-1 (GLP-1) produced in *E*. *coli* has a long-acting therapeutic effect in type 2 diabetic mice

**DOI:** 10.1371/journal.pone.0181939

**Published:** 2017-07-27

**Authors:** Fangfang Xu, Kevin Yueju Wang, Nan Wang, Gangqiang Li, Dehu Liu

**Affiliations:** 1 Institute of Biotechnology Research, Chinese Academy of Agricultural Sciences, Beijing, China; 2 Natural Sciences Department, Northeastern State University. Broken Arrow, OK, United States of America; Max Delbruck Centrum fur Molekulare Medizin Berlin Buch, GERMANY

## Abstract

Glucagon-like peptide 1 (GLP-1) is a very potent insulinotropic hormone secreted into the blood stream after eating. Thus, it has potential to be used in therapeutic treatment of diabetes. The half-life of GLP-1, however, is very short due to its rapid cleavage by dipeptidyl peptidase IV (DPP-IV). This presents a great challenge if it is to be used as a therapeutic drug. GLP-1, like many other small peptides, is commonly produced through chemical synthesis, but is limited by cost and product quantity. In order to overcome these problems, a sequence encoding a six codon-optimized tandem repeats of modified GLP-1 was constructed and expressed in the *E*. *coli* to produce a protease-resistant protein, 6×mGLP-1. The purified recombinant 6×mGLP-1, with a yield of approximately 20 mg/L, could be digested with trypsin to obtain single peptides. The single mGLP-1 peptides significantly stimulated the proliferation of a mouse pancreatic β cell line, MIN6. The recombinant peptide also greatly improved the oral glucose tolerance test of mice, exerted a positive glucoregulatory effect, and most notably had a glucose lowering effect for as long as 16.7 hours in mice altered to create a type 2 diabetic condition and exerted a positive glucoregulatory effect in *db/db* mice. These results indicate that recombinant 6×mGLP-1 has great potential to be used as an effective and cost-efficient drug for the treatment of type 2 diabetes.

## Introduction

Diabetes is a major chronic systemic metabolic disease resulting from the dysfunction of carbohydrate metabolism due to a relative deficiency of insulin [1]. The prevalence of this disease in humans has been estimated to be 415 million worldwide and is predicted to increase to 642 million by 2040 [2]. The majority of diabetic patients have Type 2 Diabetes Mellitus, which is characterized by a combination of interrelated metabolic disorders, including continuous hyperglycemia, peripheral insulin resistance, and decreased β cell function, loss or dedifferentiation [3]. Although various treatments, such as diet, exercise, antidiabetic drugs, and subcutaneous insulin injection are available, no cure is currently available for type 2 diabetes.

Glucagon-like peptide 1 (GLP-1), secreted mainly from the proximal small intestine endocrine L cells after eating [4], is a short (30 amino acids) incretin hormone. GLP-1 has been demonstrated to have pleiotropic, therapeutic effects on diabetes, such as modulating insulin secretion in a glucose-dependent manner, enhancing insulin sensitivity, suppressing glucagon secretion, promoting pancreatic β cell proliferation, aiding the restoration of normal β cell function, limiting food intake, inhibiting gastrointestinal motility, and decreasing postprandial glycemic excursions, without the risk of hypoglycemia; thus maintaining glucose homeostasis [5–9]. These attributes make GLP-1 an ideal therapeutic candidate for the treatment of type 2 diabetes.

Unfortunately, the active forms of GLP-1, GLP-1(7–36) amide and GLP-1(7–37), have a very short physiological half-life of less than 2 minutes. This is due to the rapid cleavage of the amide bond of alanine^8^ (Ala^8^) at the N-terminal by DPP-IV, resulting in two truncated inactive forms, GLP-1(9–36) and GLP-1(9–37) [10]. Therefore, clinical applications of GLP-1 have been very limited. Efforts to improve the stability of GLP-1 in the blood stream have focused on the use of structural modifications [11], competitive inhibitors [12] and adaptive delivery systems [13]. Jomori *et al*. replaced the Ala residue in GLP-1 with serine (Ser), resulting in the modified peptide having increased resistance to DPP-IV cleavage [14]. Exenatide (exendin-4) is a potent GLP-1 receptor agonist, in the primary structure of which, glycine (Gly) residue instead of Ala residue in GLP-1, also resulted in prolonging the half-life of GLP-1 [15]. Therapeutic peptides, including GLP-1, are commonly obtained by chemical synthesis, which is expensive and produces only limited yields of active peptide. Recombinant DNA biotechnology has the potential to overcome the disadvantages associated with chemical synthesis and produces these high-yields of peptides at a low cost. Currently, bacteria, yeast, and transgenic plants have been used as bioreactors to produce recombinant human GLP-1 [16–18]. It is worth noting that small peptides, such as GLP-1, are difficult to obtain when conventional expression and purification systems are used, due to the low molecular weight of the synthesized peptide and the susceptibility of the small peptides to degradation.

In the present study, a recombinant long-acting, mutated human GLP-1 (mGLP-1) was modified to eliminate the recognition site of DPP-IV and to avoid trypsin digestion. The coding sequence was constructed to contain six tandem repeats of modified mGLP-1 (6×mGLP-1). The construct was subsequently codon-optimized and expressed in *E*. *coli* transetta (DE3). The blood glucose-lowering activity of recombinant 6×mGLP-1 was then evaluated for the treatment of type 2 diabetes.

## Materials and methods

### Experimental animals

Kunming (KM) mice and *db/db* mice at specific pathogen free (SPF) level, mouse food and bedding were purchased from Charles River Technology (Beijing, China). All animal experiments were conducted following relevant guidelines and regulations and approved by the Chinese Academy of Agriculture Sciences (CAAS) Institutional Animal Ethical and Welfare Committee (No: BRISPF-2016-03). All mice were euthanized three months after experiments by CO_2_ asphyxiation.

### Design and gene synthesis of 6×mGLP-1

**Fig 1:**
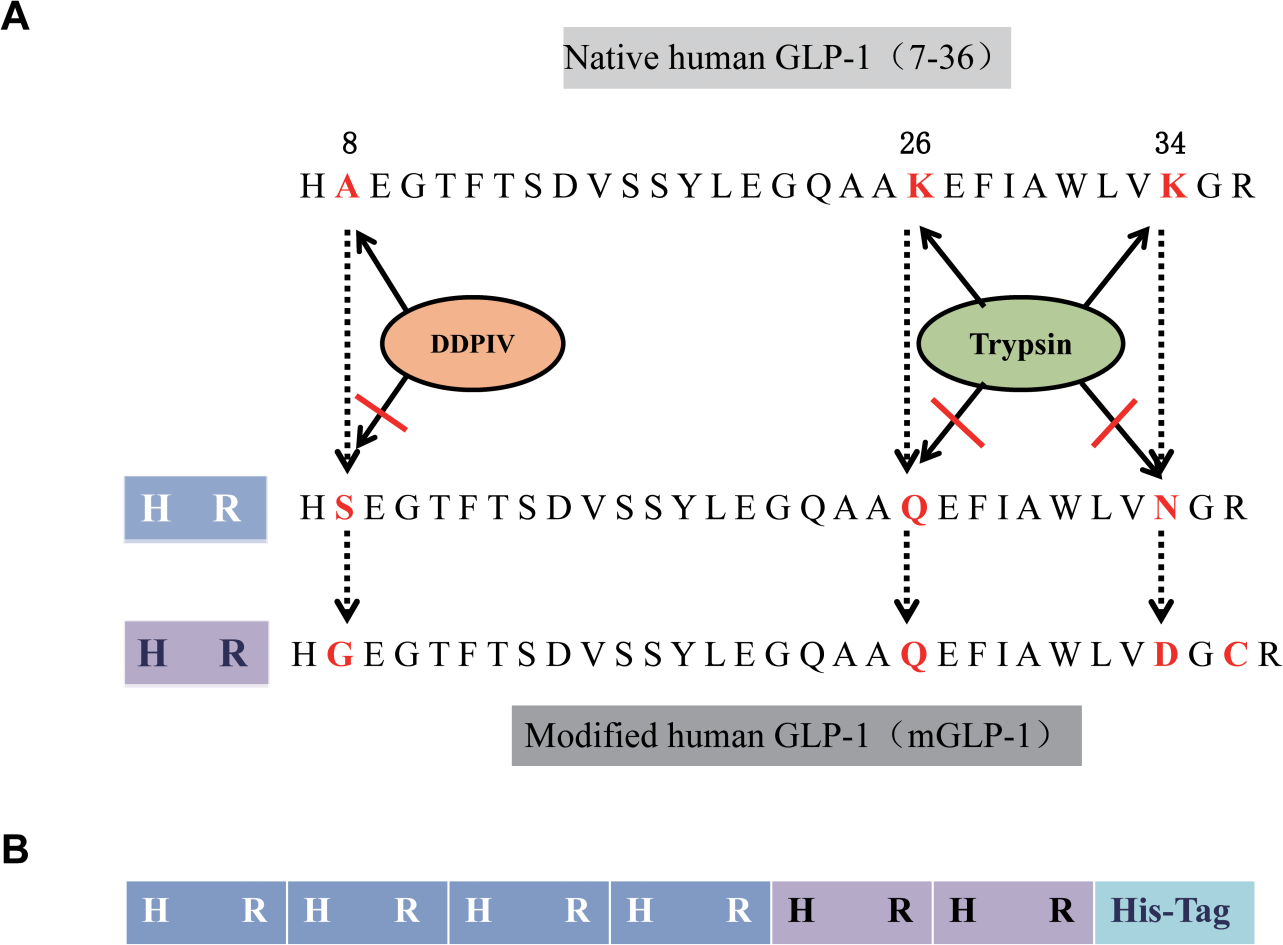
Schematic diagram the design of a protease-resistant GLP-1. (**A**) Protease resistant design of modified GLP-1. Two constructs of monomer mGLP-1 are represented in blue and purple at the left of the construct. DPPIV is in orange and trypsin enzyme is in green. Arrows indicate the cleavage sites in the native GLP-1, while blocked arrows indicate amino acid modifications (A-Ala, K-Lys, S-Ser, G-Gly, Q-Gln, N-Asn, D-Asp, C-Cys) to prevent cleavage by DPP-IV and trypsin. (**B**) Schematic diagram of a GLP-1 tandem-repeat construct (6×mGLP-1). Addition of a His-tag is indicated with light blue.

The DNA sequence of the mature active form of human GLP-1(7–36) was used as a template. Ala^8^ was mutated to Ser^8^ or Gly^8^ to prevent DPP-IV recognition and cleavage, lysine^26, 34^ (Lys^26, 34^) were mutated to glutamine^26^ (Gln^26^) and asparagine^34^ (Asn^34^) or, Gln^26^ and asparagic acid^34^ (Asp^34^) to inhibit trypsin digestion and two cysteine (Cys) residues were added to it as an attempt to prolong the half-life of the recombinant protein in the bloodstream. The modified DNA construct of GLP-1s (mGLP-1) was constructed to contain six tandem repeats of mGLP-1 genes, plus a His-tag at the C-terminus, and was named 6×mGLP-1 (**Fig 1**). After optimizing the codon sequence for expression in *E*. *coli*, the 6×mGLP-1 gene sequence was synthesized by GenScript Co., Ltd (Nanjing, China) into pUC57-6×mGLP-1 plasmid.

### Construction of the expression plasmid, pEASY-E2-6×mGLP-1

Full-length DNA of 6×mGLP-1(603bp) was obtained through PCR with pUC57-6×mGLP-1 plasmid as a PCR template. The following primer pair was used for the PCR amplification: GLP1-F: 5’-AAAGTGCACGCCACCAGACA-3’ and GLP1-R: 5’-GCCAAGCTTTTTCCTAGGTTAATG-3’. The PCR product was separated by 1% agarose gel electrophoresis, cloned into the pEASY-E2 expression vector (TransGen Biotech, Beijing, China) by TA cloning and confirmed by DNA sequencing after PCR amplification. The constructed recombinant plasmid pEASY-E2-6×mGLP-1 in *E*. *coli* trans1-T1 cloning strains was extracted and subsequently transformed into *E*. *coli* transetta (DE3) expression strain (TransGen Biotech, Beijing, China).

### Expression and confirmation of 6×mGLP-1 by SDS-PAGE and Western blot

The confirmed *E*. *coli* transetta (DE3) strain harboring pEASY-E2-6×mGLP-1 was cultured in 20 mL Luria-Bertani (LB) culture medium with 50 mg ampicillin per liter at 37°C to an OD_600_ = 0.6 ~ 0.8. Then, 1.0 mmol/L isopropy-β-D-thiogalactoside (IPTG) was added into the culture medium to induce the expression of the 6×mGLP-1for approximately 6 ~ 8 hours. Harvested bacterial cells were ultra-sonicated in phosphate buffer saline (PBS) (137 mmol/L NaCl, 2.7 mmol/L KCl, 10 mmol/L Na_2_HPO_4_, 2 mmol/L KH_2_PO_4_, pH 7.4) and then centrifuged at 4°C at 12000 rpm for 10 min. The culture supernatant and insoluble fractions (20 μL) were collected for separation using sodium dodecyl sulfate-polyacrylamide gel electrophoresis (SDS-PAGE) and visualized by staining the gels with coomassie blue R-250. The *E*. *coli* transetta (DE3) strain harboring the plasmid pEASY-E2-6×mGLP-1 without IPTG induction served as the negative control. The proteins that were separated on the SDS-PAGE gels were transferred onto polyvinylidene fluoride (PVDF) membranes (Sigma-Aldrich, St. Louis, MO, United States). Western blotting analysis was performed using mouse anti-GLP-1 monoclonal antibody (1.00 mg/mL, ab23472, Abcam Ltd., Cambridge, United Kingdom) as the primary antibody and rabbit anti-mouse immunoglobulin G-alkaline phosphatase (IgG-AP) as the secondary antibody (1.00 mg/mL, ab6729, Abcam Ltd, Cambridge, United Kingdom), with a dilution of 1: 2000 and 1: 3000, respectively. The immuno-labeled bands were visualized with a 5-bromo-4-chloro-3-indolyl phosphatase/nitro-blue tetrazolium thloride (BCIP/NBT)solution according to the manufacturer’s instructions (VWR, Radnor, PA, United States).

### Large scale production and purification of 6×mGLP-1

A single-colony transetta (DE3) strain with pEASY-E2-6×mGLP-1 plasmid was cultured in 500 mL LB culture with 50 μg/mL ampicillin at 37°C. The cultures were then induced by the addition of 0.3 mmol/L IPTG for 8 hours. The cells were then harvested by centrifugation at 12,000 rpm for 5 min at 4°C and pelleted cells were disrupted by ultra-sonication after re-suspension in PBS buffer (137 mmol/L NaCl, 2.7 mmol/L KCl, 10 mmol/L Na_2_HPO_4_, 2 mmol/L KH_2_PO_4_, pH 7.4). After centrifugation at 15000 rpm for 30 min at 4°C, 6×mGLP-1-His fusion protein, in the form of inclusion bodies, was collected from the crude cell lysate, dissolved in 8 mol/L urea solution, and further purified though a His-tagged affinity chromatography column of nickel-nitrilotriacetic acid (Ni-NTA) bind resin (Qiagen, Hilden, Germany). The recombinant protein was then subjected to dialysis for renaturation, and then lyophilized. Samples of the purified 6×mGLP-1-His fusion protein were kept at 4°C for further use.

Reversed-phase high performance liquid chromatography (HPLC) was used to confirm the purity of 6×mGLP-1, and monitor the digestion of 6×mGLP-1 by trypsin. The HPLC assay was performed on C_18_ column (250×4.6 mm, I.D.S-5 μm, 12 nm, YMC-Pack ODS-A, Germany) using LC-2010A/C chromatographic instrument (Shimadzu, Japan), at ambient temperature with a mobile phase of acetonitrile/water (90: 10, v/v) at a flow rate of 1.0 mL/min. Trifluoroacetic acid (TFA, 0.1%) was added into the water. Gradient assay was lasted for 20 min. All samples were detected at 280 nm. The blank, PBS (pH 7.4), trypsin and commercial GLP-1 were used as controls.

### Analysis of 6×mGLP-1 digestion by trypsin *in vitro*

Purified 6×mGLP-1 (10 mg/mL) was incubated with trypsin enzyme (4 mg/mL) (Biodee, Beijing, China) in physiological saline for 5, 10, 15, 20, 25, 30, 45, 60, 90, and 120 min at 37°C. Trypsin and recombinant 6×mGLP-1 alone served as negative controls. A commercial GLP-1 standard (PeproTech, Rock Hill, NJ, United States) was used as the positive control. The incubated samples (20 μL) at the various time points were mixed with SDS-loading buffer respectively and then boiled for 10 min. The digestion assay was analyzed by SDS-PAGE, and visualized with coomassie blue R-250 and silver staining, respectively.

### Biological activity of the recombinant 6×mGLP-1 *in vitro*

Based on the method described by Brandsma *et al*. [18], with minor modifications, a mouse pancreatic tumor β cell line (MIN6) (iCell Bioscience Inc, Shanghai, China) was cultured and maintained in Roswell Park Memorial Institute (RPMI) 1640 medium containing 10% (v/v) fetal bovine serum, 100 U/mL penicillin, 0.1 mg/mL streptomycin and 50 μmol/L 2-mercaptoethanol at 37°C in an incubator with 5% CO_2_ and 95% humidified air. MIN6 cells were cultured to a density of 6×10^4^ cells/mL, then seeded into a 96-well flat plate (200 μL per well) and re-cultured for 24 hours for use in determining the effect of recombinant 6×mGLP-1 on β cell proliferation. 10 μg/mL commercial GLP-1 standard or 6×mGLP-1 digested by trypsin (0.25%) were then added to the cell cultures, with 0.25% (w/v) trypsin-treated and Dulbecco’s phosphate buffered saline (DPBS) cultures serving as negative controls. The cultures were subsequently incubated for an additional 48 hours. Subsequently, 100 μL 0.5% (w/v) 3-(4,5-dimethylthiazol-2-yl)-2,5-diphenyl-2H-tetrazolium bromide (MTT) per well was added to each sample and further incubated for another 4 hours on a shaker at 37°C, after which 100 μL dimethylsulfoxide (DMSO) was added to each well. The absorbance at 490 nm was then measured in each well in a microplate reader. Six biological replicates were used for each sample treatment and the experiment was repeated twice.

### Oral glucose tolerance test

The oral glucose tolerance test (OGTT) was performed according to Kong *et al*. [19] with minor modifications. Sixteen, 8-week-old male KM mice, weighing 46.06 ± 1.66 g, were fasted overnight and divided into control and test groups (n = 8 per group). Each group was administered 2 g/kg body weight glucose into their stomach via oral administration with a proper gavage needle. The test group was simultaneously administrated, via intraperitoneal injection, a 1.0 mL dose of 0.1 mg/mL 6×mGLP-1 dissolved in physiological saline. The control group was administered an equivalent amount of physiological saline. Blood samples were taken from the tail vein of each mouse at 0, 10, 30, 60 and 120 min, and blood glucose levels were measured with a blood-glucose meter (Sinocare, Changsha, China).

### Blood glucose-lowering effect of recombinant 6×mGLP-1 on type 2 diabetic mice models

Fifteen KM mice were injected intraperitoneally with a 30 mg/kg body weight dose of streptozotocin (STZ, Sigma-Aldrich, St. Louis, MO, United States) dissolved in citrate buffer (0.1 mol/L citric acid, 0.1 mol/L sodium citrate, pH 4.2) for 2 ~ 4 consecutive days to selectively damage pancreatic β cells. In addition, they were also administered a high-fat diet (crude protein content ≥22%, crude fat ≥4.5%, and about 4000 kcal/kg calories) for one month in order to establish a stable hyperglycemic type 2 diabetic condition. Another fifteen age-, sex- and body weight-matched KM mice were given equivalent amount citrate buffer and fed with standard food as control. Mice were housed in cages with 5 mice/cage and given free access to high-fat food and water (23 ± 2°C). A 45 ± 5% relative humidity was maintained and the mice were subjected to a 12/12 h light/dark cycle. After approximately one month, when blood glucose levels in the STZ-induced mice were as high as 20.80 ± 2.21 mmol/L, OGTT was performed as described above (n = 15).

Fourteen STZ-induced type 2 diabetic mice were selected and randomly divided into 2 groups (n = 7 per group). The mice in both groups were fasted until the end of the experiment but given a normal water supply. The test group was injected intraperitoneally in the lower, left portion of the abdominal cavity with 0.1 mg/kg 6×mGLP-1 (1.0 mL) dissolved in physiological saline while the control group was injected with an equivalent dose of physiological saline. Blood samples were obtained from the tail vein of each mouse and glucose levels at various time points were measured with a blood glucose monitor.

In another experiment, twelve *db/db* mice of 9 week-old bought from Beijing Vital River Laboratory Animal Technology Co., Ltd. were given free access to standard food and water (23 ± 2°C). A 45 ± 5% relative humidity was maintained and the mice were subjected to a 12/12 h light/dark cycle. One week later, they were divided into 2 groups randomly. In one group, mice were given 0.1 mg/mL 6×mGLP-1 (1.0 mL) via oral gavage, while mice in the other group were given the same volume of physiological saline (n = 6). Mice were all fasted but given free access to water. Blood samples were collected from the tail tip at various time points and measured using a glucose monitor.

### Statistical analysis

The presented data are means ± standard deviation (SD). Significant differences between control and test groups were determined by a Student’s *t*-test or one-way analysis of variance (ANOVA) followed by *post-hoc* hypothesis tests, where appropriate, using SPSS version 19.0 software. In all statistical analyses, a *P* value < 0.05 was considered to indicate a statistically significant difference.

## Results and discussion

### Protease resistant design of 6×mGLP-1

In order to overcome the short half-life of GLP-1 in the bloodstream, studies have mainly focused on identifying and developing DPP-IV inhibitors [20, 21], modifying the structure of GLP-1, or searching for structural analogues that are more resistant to DPPIV cleavage [22]. Based on these studies, and the functional form of native GLP-1(7–36), a modified human GLP-1 (mGLP-1) was designed and constructed by site-specific mutations that would have resistance to both DPP-IV and trypsin cleavage. This included replacing the enzyme-susceptible Ala at 8 position and Lys at the 26 and 34 position. Ala^8^ was mutated to code for Ser^8^ or Gly^8^, and Lys^26, 34^ were substituted with Gln^26^, Asn^34^ or Gsn^26^, Asp^34^. These structural modifications have been confirmed to exert complete resistance to DPP-IV induced cleavage of GLP-1 and trypsin degradation [23–26]. In the present study, these point-modifications were placed into a GLP-1 construct consisting of six mGLP-1 coding sequences present as a tandem repeat (6×mGLP-1) in order to obtain a longer half-life once the mGLP-1 was present in the bloodstream. Furthermore, two Cys residues were added before the last amino acid, arginine (Arg), in order to enhance the stability of mGLP-1 in the bloodstream by the formation of disulfide bonds (**Fig 1A**).

Additionally, each amino acid sequence of mGLP-1 started with a His and ended with Arg, and an extra Arg was added at the N-terminal of 6×mGLP-1 (**Fig 1B**), so that the tandemly repeated six mGLP-1 construct would be cleaved by trypsin into six separate mGLP-1monomers without any amino acids from the expression vector or His-tag. If properly functioning, this design would reduce production costs and potentially overcome the present limitation on the clinical use of GLP-1.

### Expression and purification of recombinant 6×mGLP-1

**Fig 2:**
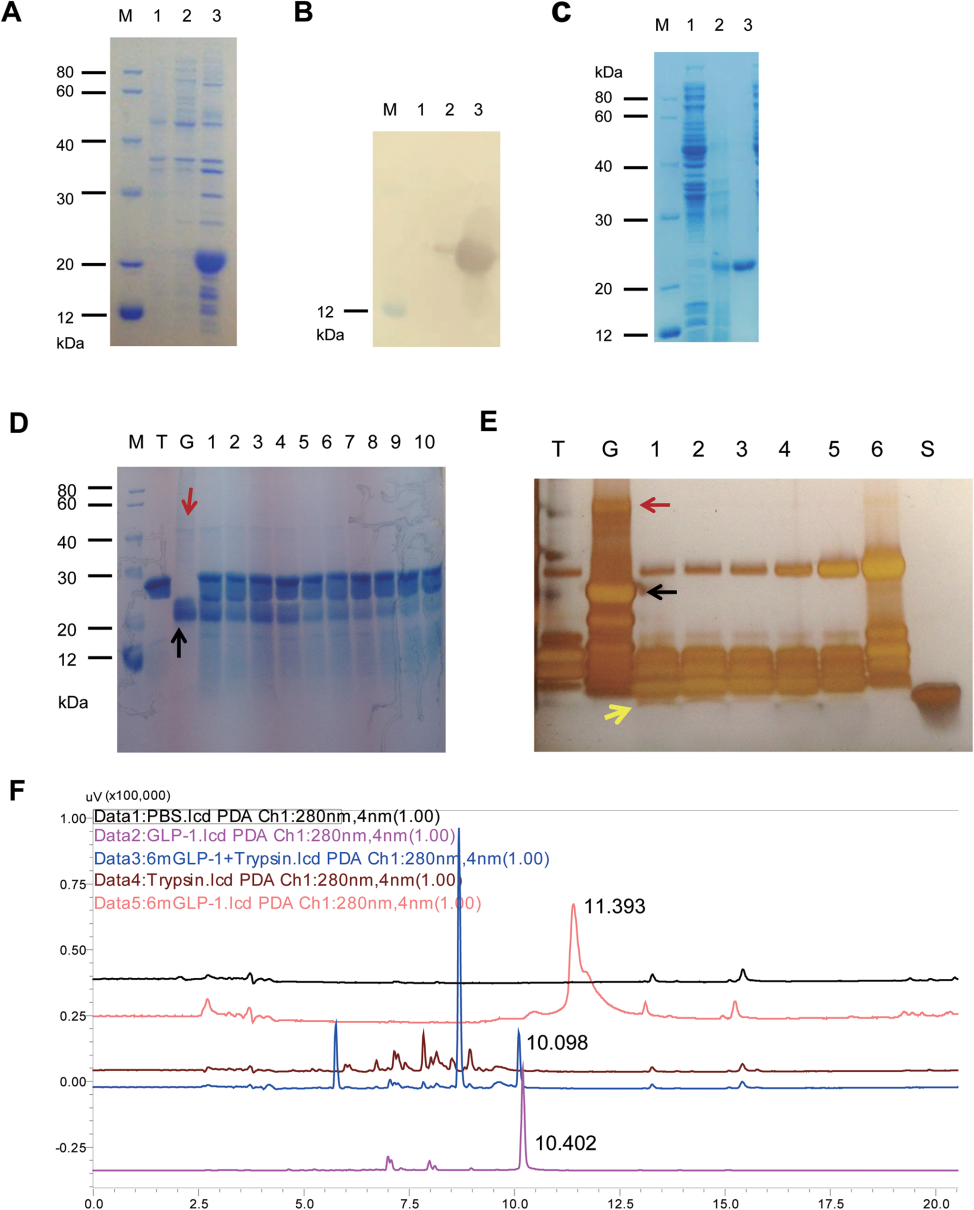
Expression confirmation, purification analysis, and digestion of 6×mGLP-1 by trypsin. (**A**) SDS-PAGE gels stained with coomassie blue R-250. (**B**) Immunoblot analysis using anti-GLP-1 antibody to the 6×mGLP-1 recombinant protein obtained from ultrasonicated cell lysate. M: protein marker, Lane 1: the bacterial cell lysate without induction by IPTG, Lane 2: supernatant of the bacterial cell lysate induced by IPTG, Lane 3: insoluble portion of the bacterial cell lysate induced by IPTG. (**C**) Analysis of the purification of 6×mGLP-1-His tag fusion protein. M: protein marker, Lane 1: unbound eluate from the Ni-NTA affinity chromatography column using crude protein extracts from pEASY-E2-6×mGLP-1/transetta (DE3) cells induced by IPTG, Lane 2: bound eluate, Lane 3: final purified 6×mGLP-1. (**D**) Proteolytic analysis of 6×mGLP-1-His tag fusion protein by SDS-PAGE stained with coomassie blue R-250. M: protein MW marker, T: Trypsin enzyme, G: 6×mGLP-1, Lane 1–10: trypsin digestion of 6×mGLP-1 for 5, 10, 15, 20, 25, 30, 45, 60, 90, 120 min at 37°C, respectively. The dimer form of 6×mGLP-1 is indicated in red arrow and 6×mGLP-1 in black arrow. (**E**) Proteolytic analysis of 6×mGLP-1-His tag fusion protein by SDS-PAGE visualized with silver staining. T: Trypsin enzyme, G: 6×mGLP-1, S: native human GLP-1 synthesized chemically as standard. Lane 1–6: trypsin digestion of 6×mGLP-1 for 15, 30, 45, 60, 90, 120 min at 37°C, respectively. The dimer form of 6×mGLP-1 is indicated in red arrow, 6×mGLP-1 in black arrow, and yellow arrow represented that 6×mGLP-1 was cleaved into monomers.

The 6×mGLP-1 codon sequence was optimized for expression in *E*.*coli* strain, transetta (DE3). As shown in the SDS-PAGE (**Fig 2A**) and immunoblotting (western blotting, **Fig 2B**), the target protein was expressed abundantly in the form of an insoluble inclusion body. The molecular weight (MW) of designed 6×mGLP-1 was slightly more than 20 kDa (**Fig 2**), which is consistent with its predicted size of approximately 22 kDa. High-quality 6×mGLP-1 recombinant protein was recovered with through a series of proficient steps, including denaturation and refolding in Ni-NTA affinity chromagraphy and during the process of dialysis. SDS-PAGE analysis of the reconstituted sample of 6×mGLP-1 indicated that a large amount of highly pure recombinant 6×mGLP-1 protein was obtained (**Fig 2C**). The purification result of HPLC analysis also showed that the purity of 6×mGLP-1 was as high as 100%, and the retention time of the peak was 11.393 min (Fig 2F). The estimated yield was 20 mg/L.

### Digestion analysis of 6×mGLP-1 by protease in vitro

The purified recombinant 6×mGLP-1 formed dimers (possibly because of additional disulfide bond) that could be cut by trypsin into single stable mGLP-1s with physiological activity as expected was observed to be more stable against to trypsin enzyme degradation. Consistent with Jomori and his colleagues’ findings [14], the individual mGLP-1 monomers, however, exhibited strong resistance to trypsin digestion, which was directly verified by conducting an *in vitro* protease digestion analysis assay. Coomassie-stained SDS-PAGE gels indicated that rapid *in vitro* cleavage (within 25 min) of the purified recombinant 6×mGLP-1 by trypsin resulted in small fragments (**Fig 2D**). By 120 min, very little intact 6×mGLP-1 was present (**Fig 2D**). These results demonstrate that 6×mGLP-1 can be cleaved by trypsin. Furthermore, although the 6×mGLP-1 polypeptide could be cut into single mGLP-1 fragments by trypsin *in vitro*, the mGLP-1 monomer (**Fig 2E**, yellow arrow) was resistant to any further digestion by trypsin. As shown in **Fig 2D**, 6×mGLP-1 appeared to form a dimer with a molecular weight above 40 kDa (red arrow), which was corresponding to the theoretical molecular weight of 44 kDa for the dimer. The dimer appeared more stable than the monomer form of 6×mGLP-1, because it only began to decrease 45 min after the trypsin digestion. Aberle *et al*. [27], reported that inter-disulfide bonds represent a means to stabilize peptides or protein. Therefore, we speculated that the formation of the dimer was due to the additional Cys residues that were inserted into 6×mGLP-1. This insertion was expected to prolong the half-life of 6×mGLP-1 in the bloodstream and allow for the glucose lowering effect to persist in the bloodstream for a longer period of time [27, 28].

HPLC was also used to monitor the digestion of 6×mGLP-1 by trypsin, as shown in **Fig 2F.** For purified 6×mGLP-1 only, the retention time of peak was at 11.393 min; however, the retention time of peak of 6×mGLP-1 incubated with trypsin for 2 hours at 11.393 min was missing, and one major peak was at 10.098 min which was consistent with that of GLP-1 commercial standard, 10.402 min. We speculated that 6×mGLP-1 was hydrolyzed to monomer GLP-1s when it was incubated with trypsin, which were more stable against to trypsin enzyme degradation. Trypsin was also activated at 37°C in PBS (pH 7.4). Dynamic changes occurred both in trypsin solution and the reaction solution of trypsin and 6×mGLP-1, corresponding to the results of HPLC analysis. There were two other major peaks at 5.751 and 8.676 min were observed in the reaction solution. Presumably some other forms of products were produced from 6×mGLP-1 and trypsin incubation.

### Promoting β cell proliferation

**Fig 3:**
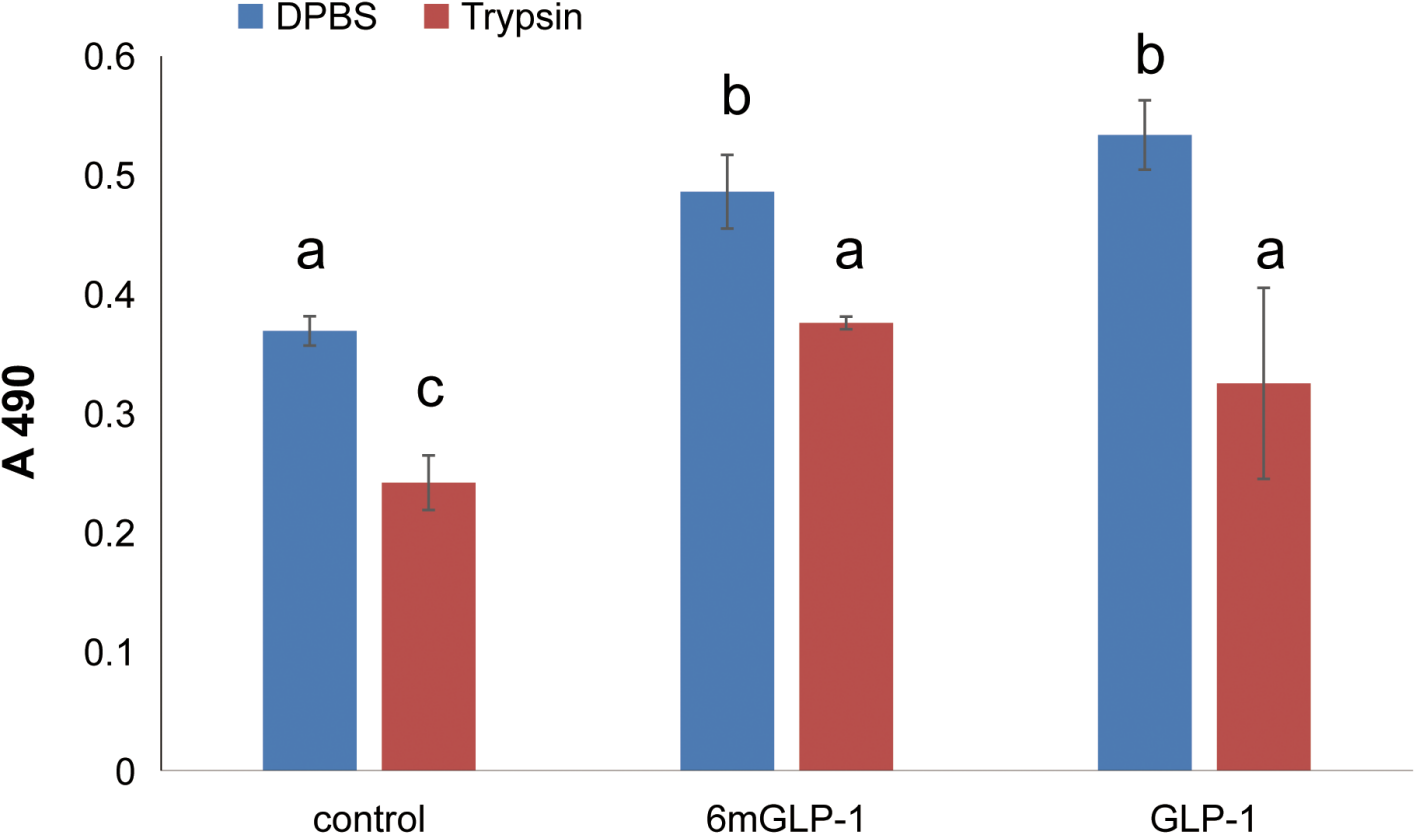
Effect of 6×mGLP-1 monomers on the proliferation of a mouse pancreatic β cell line. MIN6 cells were cultured to a density of 6×10^4^ cells/mL, then seeded into a 96-well flat plate (200 μL per well) and re-cultured for 24 hours. Control: cells treated with Dulbecco's phosphate buffered saline solution; Trypsin: cells treated with 0.25% trypsin; Trypsin + 6×mGLP-1: 10 μg/mL 6×mGLP-1 digested by 0.25% trypsin; GLP-1 standard: chemically synthesized native human GLP-1; Trypsin + GLP-1: 10 μg/mL GLP-1 digested by 0.25% trypsin. Cell density, measured as optical density at A490, was determined 48 h after the addition of the test compounds. The data represent the mean ± standard deviation (n = 6). The experiment was repeated three times and the results were statistically analyzed using one-way ANOVA method following Tukey *post-hoc* hypothesis test. The symbols a, b, and c refer to the significance level, and different symbol means statistical significance (at 5%, 1%, and 0.1% level) between treatments, or else, no significance.

One of the main functions of native GLP-1 in humans is to promote the proliferation of pancreatic β cells. Therefore, recombinant 6×mGLP-1 was assessed for this ability by using a mouse pancreatic β cell line, MIN6. The 6×mGLP-1 was digested by trypsin into GLP-1 monomers to exhibit activity. Proliferation of pancreatic β cells was promoted by both 6×mGLP-1 and 6×mGLP-1 digested with trypsin as well as the GLP-1 synthesized chemically as standard. Although 0.25% (w/v) trypsin had a significant negative effect (*P =* 0.002) on cell proliferation compared to DPBS-treated cells, relative to trypsin-treated cells, the mixture containing 0.25% (w/v) trypsin and 10 μg/mL 6×mGLP-1 compensated for the negative impact of the trypsin and actually showed a significant promotion of cell proliferation (*P =* 0.001). The synthesized native human GLP-1 standard exhibited the same extent promotion effect as 6×mGLP-1(**Fig 3**). The results do suggest, however, that 6×mGLP-1 as a whole or digested by trypsin *in vitro* could have a positive effect on the proliferation of mouse pancreatic β cells. This was consistent with Brandsma and his colleagues’ studies [18], in which 10×GLP-1 was proved to increase insulin release from MIN6 cells in the presence of 10 mmol/L glucose, although with reduced activity in contrast with commercial GLP-1 standard. After 48 h, the cell culture was also collected to assess the integrity of 6×mGLP-1 by western blotting analysis.

### Amelioration of glucose tolerance in normal KM mice

**Fig 4:**
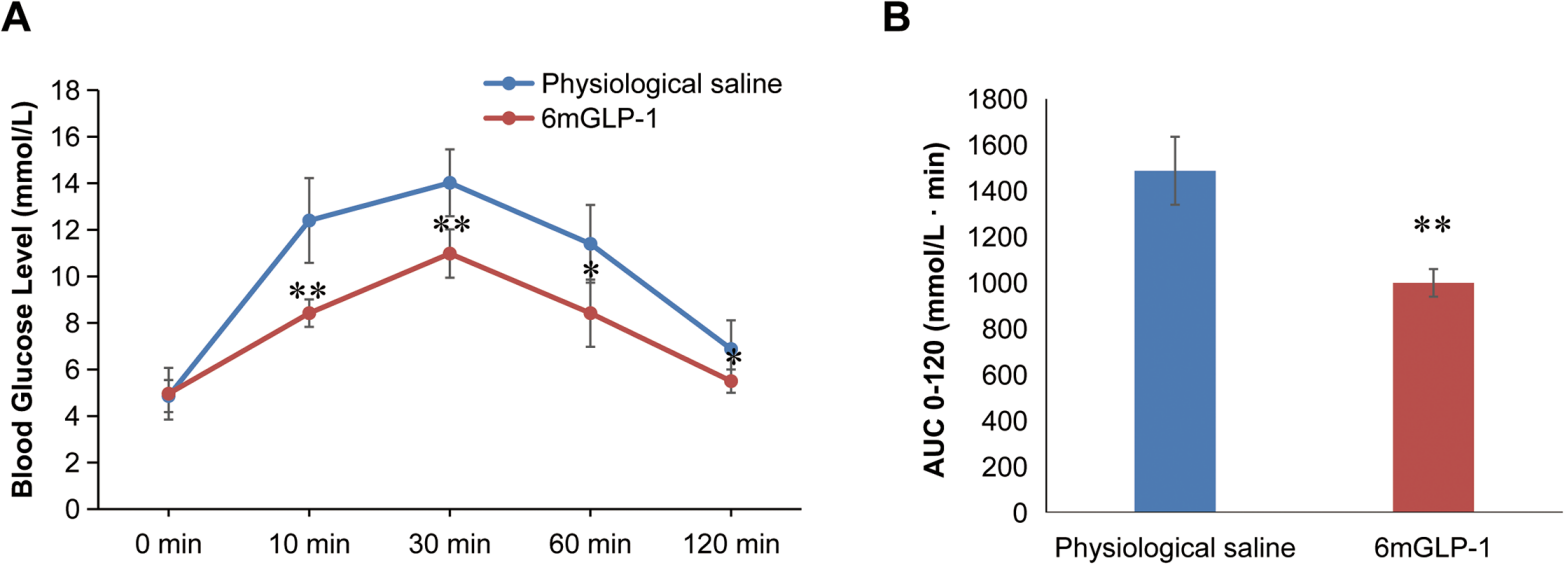
Oral glucose tolerance test of recombinant 6×mGLP-1. (**A**) Blood glucose levels of physiological saline- and 6×mGLP-1-treated mice over a 120-min time course (n = 6). (**B**) The area under the blood glucose level curve (AUC) of (**A**) from 0–120 min. This experiment was repeated twice and the results were statistically analyzed using Student’s *t*-test. * and ** represents significance differences between the two groups at 5% and 1% level, respectively.

It is widely accepted that GLP-1 plays potent roles in glucose homeostasis, and similarly our recombinant 6×mGLP-1 was proved to exert a blood glucose-lowering effect on normal KM mice when administered intraperitoneally. OGTT in mice showed that plasma glucose level of the 6×mGLP-1-treated mice was 10.98 ± 1.04 mmol/L at its peak, which was significantly lower (*P =* 0.005) than the control group which had a peak plasma glucose level of 14.02 ± 1.44 mmol/L (**Fig 4A**). The difference between the control and test groups of mice in plasma glucose levels persisted even as the glucose levels began to decrease in both groups. At 120 minutes, when the blood glucose level in the 6×mGLP-1 test group returned to normal (5.50 ± 0.50 mmol/L), the control group still had a glucose level of 6.88 ± 1.23 mmol/L (**Fig 4A**). The difference between the two groups was statistically significant (*P =* 0.049). Furthermore, the area under the curve (AUC) of 6×mGLP-1-administrated mice was significantly less (*P =* 0.002) than the AUC of mice injected with physiological saline (**Fig 4B**). These results demonstrated that purified recombinant 6×mGLP-1 can significantly improve the oral glucose tolerance of mice.

### Blood glucose-lowering activity of 6×mGLP-1 in type 2 diabetic mice models

**Fig 5:**
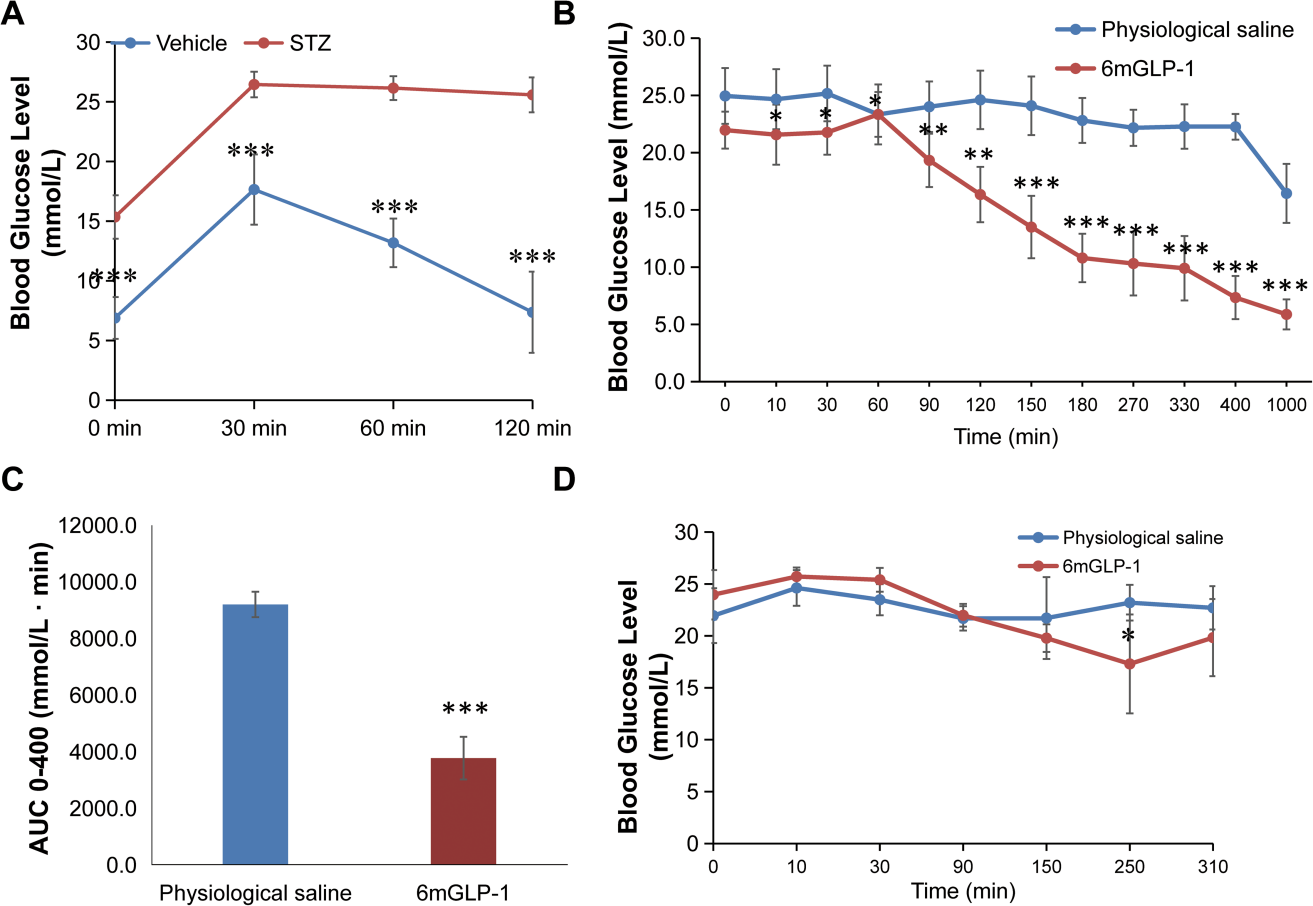
Glucoregulatory effect of recombinant 6×mGLP-1 in type 2 diabetic mice models. (**A**) Oral glucose tolerance test of STZ-induced KM mice, 6 weeks after STZ injection (n = 15). (**B**) Therapeutic effect of 6×mGLP-1 on STZ-induced type 2 diabetic mice (n = 7). Blood glucose levels were measured over a 1000-min time period. (**C**) The area under the blood glucose level curve (AUC) of (**B**) from 0–400 min. (**D**) Blood glucose-lowering effect of recombinant 6×mGLP-1 in *db/db* mice (n = 6). 6×mGLP-1 was administrated into mice via oral gavage. All experiments were repeated twice and the results were statistically analyzed using Student’s *t*-test. *, ** and *** represents significant differences between the control and test groups at the 5%, 1%, and 0.1% level, respectively.

STZ-induced type 2 diabetic mice were used to evaluated the biological activity of 6×mGLP-1. The blood glucose concentration of STZ-induced mice fed with a high fat diet was stably 21.03 ± 1.51 mmol/L five weeks after the STZ-induction began. This level was much higher than the glucose levels (8.10 ± 0.88 mmol/L) in mice that were fed a normal diet. The difference between the glucose levels in the STZ-induced and normal mice was highly significant (*P =* 4.34E-04). STZ-induced mice also showed impaired glucose tolerance compared to mice in control group (**Fig 5A**).

STZ-induced type 2 diabetic mice were fasted but with normal water supply when separately treated with physiological saline and 6×mGLP-1. The plasma glucose level in the control group of mice remained high for at least 400 min, and then began to decrease, possibly due to hunger (at 1000 min with a blood glucose level of 16.44 ± 2.58 mmol/L, comparable with 15.35 ± 1.82 mmol/L when fasted overnight in OGTT, as shown in **Fig 5A**); while the blood glucose level in the test group decreased gradually over a period of 60–1,000 min (**Fig 5B**). These data demonstrated that the effect of 6×mGLP-1 occurring over an extended period of time suggested its ability to gradually lower plasma glucose, without the risk of hypoglycemia (5.87 ± 1.31 mmol/L glucose concentration at 1,000 min, nearly 16.7 hours, much longer than our previous monomer mGLP-1 [29]). A significant difference (*P =* 0.026) between the two groups in blood glucose levels developed rapidly within 10 min and the separation lasted throughout the entire experiment. Notably, the level of the significant difference between the test and control groups increased with the duration of the experiment (*P*<0.05 at 0–60 min, *P* <0.01 at 60–120 min, and *P*<0.001 at 150–1,000 min). These differences may reflect the gradual breakdown of 6×mGLP-1 into mGLP-1 monomers by endogenous trypsin in the mice, allowing for extended activity of the recombinant protein. The AUC of the control group of mice from 0 to 400 minutes was significantly higher (*P* = 8.5E-06) than the AUC of the test group (**Fig 5C**).

*db/db* mice were also used to evaluated the biological activity of 6×mGLP-1. The blood glucose level of oral 6×mGLP-1-treated mice began to decrease at 90 min, and didn’t show any statistical difference until 250min (*P =* 0.017), with glucose concentration 17.30 ± 4.77 mmol/L. Then it began to rise (**Fig 5D**). Although the route of giving 6×mGLP-1 via oral gavage made it function later possibly resulted from digestion of 6×mGLP-1 into monomers in the gastrointestinal tract at first before absorption into the blood circulation, and last no longer than 4 hours compared with i.p. injection, it put great confidence in chronic treatment of *db/db* mice via oral administration next.

GLP-1 was confirmed to exert pleiotropic therapeutic effects on diabetes. GLP-1 analogue therapy has been considered a promising therapeutic option for type 2 diabetes mellitus. Preclinical observations suggested its direct renoprotective effects in the setting of hypertension, diabetic and nondiabetic nephropathy, independent of the glucose-lowering effects [30]. As to GLP-1 receptor agonists, attention has been paid on chronic kidney disease primarily caused by hypertension and diabetes. Hence, potential non-glycemia related effects of 6×mGLP-1 will be studied and evaluated in further studies, especially in diabetic renal impairment.

In summary, we modified GLP-1 to prolong the half-life of GLP-1. Six codon-optimized tandem repeats of modified GLP-1, 6×mGLP-1, was successfully expressed in *E*.*coli*. Recombinant 6×mGLP-1 that could be cut by trypsin into single stable mGLP-1s with physiological activity as expected was proved to be more stable against to trypsin enzyme degradation. The use of 6×mGLP-1 lowered glucose and might have an antidiabetic effect and this finding could encourage clinical trials using 6×mGLP-1 in type 2 diabetic patients. Utilization of this protein could provide a potential cost-effective method for GLP-1 therapy and warrants additional research in the future.

## Supporting information

S1 Table A list of abbreviations in alphabetical order. (XLSX)
